# Real-World Outcomes of Second-Line Chemotherapy in Metastatic Urothelial Carcinoma

**DOI:** 10.3390/curroncol33090529

**Published:** 2026-09-02

**Authors:** İlkay Çıtakkul, Hayati Arvas, Mert Karaoğlan, Bahadır Köylü, Nazan Demir, Gözde Balkaya Aykut, Elif Şahin, Mesut Yılmaz, Zuhat Urakçı, Duygu Bayır Garbioğlu, Fatih Selçukbiricik, Ece Baydar, Beşire Nurdan Tazebay, Melike Yazıcı, Yasemin Bakkal Temi, Devrim Çabuk, Kazım Uygun, Umut Kefeli

**Affiliations:** 1 Department of Internal Medicine, Division of Medical Oncology, Faculty of Medicine, Kocaeli University, Kocaeli 41001, Türkiye; ecebaydar@gmail.com (E.B.); besiretazebayy@gmail.com (B.N.T.); drmelikeyzc@gmail.com (M.Y.); yasemin.temi@kocaeli.edu.tr (Y.B.T.); devrimcabuk@yahoo.com (D.Ç.); kzuygun@hotmail.com (K.U.); ukefeli@yahoo.com (U.K.); 2 Department of Internal Medicine, Division of Medical Oncology, Faculty of Medicine, Dicle University, Diyarbakır 21280, Türkiye; hayatiarvas65@gmail.com (H.A.); zuhat.urakci@dicle.edu.tr (Z.U.); 3 Department of Medical Oncology, Faculty of Medicine, Eskişehir Osmangazi University, Eskişehir 26480, Türkiye; mertkaraoglann@gmail.com (M.K.); duygubayir@hotmail.com (D.B.G.); 4 Department of Internal Medicine, Division of Medical Oncology, School of Medicine, Koç University, Istanbul 34450, Türkiye; bahadirkoylu@hotmail.com (B.K.); ndemir@kuh.ku.edu.tr (N.D.); fselcukbiricik@ku.edu.tr (F.S.); 5 Division of Medical Oncology, Ümraniye Training and Research Hospital, Istanbul 34764, Türkiye; gozdebalkayaaykut@gmail.com; 6 Division of Medical Oncology, Kocaeli City Hospital, Kocaeli 41060, Türkiye; 7 Department of Medical Oncology, Faculty of Medicine, Sakarya University, Sakarya 54050, Türkiye; mstsnm08@gmail.com

**Keywords:** bladder cancer, chemotherapy, overall survival, propensity score, second-line treatment, urothelial cancer

## Abstract

Second-line chemotherapy is widely used in metastatic urothelial carcinoma after progression on first-line platinum-based therapy, but its independent contribution to survival, as opposed to selection of healthier patients, remains unclear. In this multicenter retrospective cohort of 142 patients treated with first-line platinum-based chemotherapy across seven Turkish centers, overall survival (OS) from first-line progression was compared between patients who received second-line chemotherapy (*n* = 80) and those who did not (*n* = 62), using multivariable Cox regression, inverse probability of treatment weighting (IPTW), propensity-score matching, landmark analysis, and a time-dependent Cox model. Median OS was 7.4 versus 4.7 months (log-rank *p* = 0.064). Second-line chemotherapy was independently associated with improved OS on multivariable analysis (adjusted hazard ratio [aHR] 0.620; 95% confidence interval [CI] 0.423–0.907; *p* = 0.014); Eastern Cooperative Oncology Group (ECOG) performance status ≥ 2 (aHR 3.881; *p* = 0.001) and lower albumin (aHR 0.671; *p* = 0.018) were also independent predictors. The association remained significant after IPTW (HR 0.648; *p* = 0.025) and after a time-dependent Cox model (HR 0.632; *p* = 0.019), and was unchanged in ECOG-restricted and Bellmunt-adjusted analyses (*p* = 0.008, *p* = 0.029); it narrowly missed significance after propensity-score matching (HR 0.645; *p* = 0.051) and did not reach significance in the 3-month landmark analysis (HR 0.743; *p* = 0.180). Power was limited (~49%). In a time-dependent Cox model—the analysis least susceptible to immortal-time bias, as it retains the full cohort and classifies pre-treatment person-time as unexposed—second-line chemotherapy remained independently associated with improved OS (HR 0.632; *p* = 0.019), closely consistent with the primary multivariable estimate. The conventional Cox, IPTW, and propensity-score-matched analyses, which treat second-line receipt as a baseline exposure, were directionally concordant but share a common time-related bias and are therefore not independent confirmations. ECOG performance status was a consistent predictor throughout.

## Simple Summary

In advanced bladder cancer, chemotherapy given after the first treatment stops working is widely used, but whether it truly extends life is unclear, because patients who receive it tend to be healthier to begin with. We studied 142 patients from seven Turkish hospitals, comparing survival between those who did and did not receive this second-line chemotherapy, using several statistical methods designed to separate a true treatment effect from selection effects, including one that specifically accounts for the time patients must survive to receive treatment. Most methods, including the time-dependent Cox model that accounts for treatment timing, showed a significant survival benefit; two others, both based on smaller subgroups, did not confirm this as clearly, so some uncertainty remains. Performance status, how well a patient functions day-to-day, was the strongest, most reliable predictor of survival throughout. These findings support a real, if modest, benefit from second-line chemotherapy here, while underscoring the value of newer agents where accessible.

## 1. Introduction

Urothelial carcinoma is one of the most common malignancies of the urinary tract and a frequent cause of cancer-related death worldwide, occurring approximately three to four times more often in men than in women [1,2]. Advanced age and tobacco smoking are the principal risk factors. Although a substantial proportion of patients present with localized disease amenable to curative treatment, recurrence is common, and a significant number of patients ultimately develop metastatic disease. Metastatic urothelial carcinoma carries a poor prognosis, with a historical 5-year overall survival rate of less than 20% [3].

For several decades, platinum-based combination chemotherapy—most commonly gemcitabine combined with cisplatin or, in cisplatin-ineligible patients, carboplatin—has represented the standard first-line treatment for metastatic disease. Despite objective response rates that may exceed 40%, these responses are typically short-lived, and the median overall survival with platinum-based regimens has remained limited to approximately 14 to 15 months, and shorter still in cisplatin-ineligible patients [4,5,6]. A large proportion of patients experience disease progression within the first year, at which point subsequent-line treatment options become relevant.

Historically, options after progression on first-line platinum-based chemotherapy have been limited and of modest benefit. In the only randomized phase III trial in this setting, vinflunine plus best supportive care provided a median overall survival of 6.9 months versus 4.6 months with best supportive care alone, a difference that reached statistical significance only in the eligible population and translated into an objective response rate below 10% [7]. Taxanes such as docetaxel and paclitaxel are widely used off-label on the basis of phase II data showing similarly modest activity, and no universal consensus exists regarding the optimal salvage regimen [8,9,10]. The subsequent-line landscape was then transformed by the immune checkpoint inhibitor pembrolizumab, which demonstrated superiority over conventional chemotherapy in the KEYNOTE-045 trial [11,12]; by the antibody–drug conjugates enfortumab vedotin [13] and sacituzumab govitecan [14]; and by targeted therapy with erdafitinib in FGFR-altered tumors [15]. Enfortumab vedotin has since moved into the first-line setting in combination with pembrolizumab, based on the EV-302/KEYNOTE-A39 trial [16], and switch-maintenance immunotherapy with avelumab following first-line chemotherapy has also become an option for patients without disease progression [17,18]. However, access to these agents varies considerably across healthcare systems. In Türkiye, as in many other middle-income countries, reimbursement for immune checkpoint inhibitors and enfortumab vedotin remains limited, and in many real-world settings, including ours, a substantial proportion of patients continue to be managed with a conventional chemotherapy-based treatment sequence.

In this context, real-world data on the value of conventional second-line chemotherapy remain clinically relevant. The extent to which second-line chemotherapy independently contributes to survival—as opposed to reflecting the selection of patients with more favorable prognostic characteristics—has not been firmly established. Observational analyses of this question are prone to both measured confounding (patients selected for second-line treatment tend to have better performance status and organ function) and guarantee-time (immortal-time) bias, which arises because patients must survive from first-line progression long enough to receive second-line treatment. Established prognostic models in this disease, such as the Bajorin and Bellmunt risk scores, indicate that performance status, hemoglobin, and visceral metastasis strongly influence survival and may confound naive comparisons [19,20]. We therefore conducted a multicenter retrospective study to evaluate the association between second-line chemotherapy and overall survival, applying multivariable adjustment, propensity-score methods, and landmark analysis in parallel, and explicitly reporting where these approaches agree and where they diverge [21,22,23].

## 2. Materials and Methods

### 2.1. Study Design and Population

This was a multicenter, retrospective cohort study conducted across seven tertiary oncology centers in Türkiye: Kocaeli University Faculty of Medicine, Dicle University Faculty of Medicine, Eskişehir Osmangazi University Faculty of Medicine, Koç University Hospital, Ümraniye Training and Research Hospital, Sakarya University Faculty of Medicine, and Kocaeli City Hospital. The medical records of patients diagnosed with metastatic urothelial (bladder) carcinoma between January 2019 and October 2025 were retrospectively reviewed using a standardized case report form.

### 2.2. Eligibility Criteria

Patients aged 18 years or older with metastatic bladder cancer who had received first-line platinum-based chemotherapy were eligible. Patients with neuroendocrine histology or incomplete follow-up data were excluded, as were those who received immunotherapy, antibody–drug conjugates, targeted agents, or switch-maintenance immunotherapy at any line, to yield a cohort managed exclusively with conventional cytotoxic chemotherapy. The final analytic cohort comprised 142 patients. All patients had bladder (lower urinary tract) primary tumors; patients with upper urinary tract tumors were not included. All patients had de novo metastatic (stage IV) disease.

### 2.3. Groups and Variables

Patients were classified according to whether they received second-line chemotherapy after progression on first-line platinum-based treatment (80 patients, 56.3%) or did not (62 patients, 43.7%). Demographic characteristics (age, sex, and smoking status), Eastern Cooperative Oncology Group (ECOG) performance status and laboratory parameters (albumin, estimated glomerular filtration rate [GFR], hemoglobin, lymphocyte and platelet counts) at the time of progression, and metastatic sites (liver, lung, bone, and brain) were recorded for all patients. For patients who received second-line chemotherapy, the specific regimen administered (paclitaxel, docetaxel, methotrexate-vinblastine-doxorubicin-cisplatin (MVAC), or vinflunine) was also recorded. The Bellmunt risk score (ECOG > 0, hemoglobin < 10 g/dL, and liver metastasis; range 0–3) was derived for each patient and used as a composite prognostic covariate in a secondary model [19,20]. The primary endpoint was overall survival (OS), defined as the interval from progression on first-line therapy to death from any cause or last follow-up; this common definition applied identically to both groups.

### 2.4. Statistical Analysis

Continuous variables were expressed as median (interquartile range [IQR]) and compared using the Mann–Whitney U test; categorical variables were compared using the chi-square or Fisher’s exact test, as appropriate. OS was estimated by the Kaplan–Meier (KM) method and compared with the log-rank test.

Median potential follow-up was assessed using the reverse Kaplan–Meier method; because the proportion of censored patients was low (22/142, 15.5%), the reverse-KM median was not estimable, and the median observed follow-up among censored patients is reported instead. Cox proportional-hazards regression was used to estimate hazard ratios (HRs) with 95% confidence intervals, with the proportional-hazards assumption verified using Schoenfeld residuals. ECOG performance status was modelled as a binary variable (0–1 versus ≥2) consistent with its use as a component of the Bellmunt risk score; a sensitivity analysis restricted to patients with ECOG 0–1 was performed, and a parallel model substituting the Bellmunt risk score for its individual components was also fitted.

To evaluate the robustness of the primary multivariable estimate against confounding and immortal-time bias, four complementary analyses were performed. First, a propensity score was estimated by logistic regression (ECOG category, age, smoking, albumin, GFR, hemoglobin, and liver metastasis) and used for stabilized inverse probability of treatment weighting (IPTW) with robust standard errors, and for 1:1 nearest-neighbor propensity-score matching (caliper 0.2 standard deviation [SD] of the logit propensity score); covariate balance was assessed by standardized mean differences (SMD) and a Love plot (Supplementary Figure S1), with SMD < 0.1 considered good balance. Second, a landmark analysis was performed, restricted to patients alive at 3 months from progression and classified by ever-receipt of second-line chemotherapy; the 3-month landmark was chosen a priori as a clinically standard interval for post-progression reassessment, and 2- and 4-month landmarks were tested in sensitivity analyses to confirm that the choice of landmark did not materially affect the estimate (Supplementary Table S3). Third, a time-dependent Cox model was fitted using a counting-process (start, stop, and event) formulation, with second-line chemotherapy entered as a time-varying covariate defined by the interval from progression to second-line initiation (calculated from recorded calendar dates); this approach classifies each patient’s pre-treatment person-time as unexposed and uses the complete cohort without discarding early events, in contrast to the landmark approach.

A post hoc power calculation was performed for the observed number of events and hazard ratio. A two-sided *p* < 0.05 was considered significant. Analyses were performed using Python 3.12 (lifelines 0.30.3 and statsmodels 0.14.6 packages) and IBM SPSS Statistics for Windows, version 29.0 (IBM Corp., Armonk, NY, USA).

### 2.5. Ethics

The study was approved by the Kocaeli University Non-Interventional Clinical Research Ethics Committee (approval no. GOKAEK-2026/02/20, dated 15 January 2026; project no. 2026/38) and conducted in accordance with the Declaration of Helsinki. Given the retrospective design, the requirement for written informed consent was waived.

## 3. Results

### 3.1. Patient Characteristics

A total of 142 patients were included; 80 (56.3%) received second-line chemotherapy, and 62 (43.7%) did not. The cohort was predominantly male (85.2%), with a median age of 65 years. Groups were balanced with respect to age, sex, ECOG performance status, GFR, hemoglobin, lymphocyte and platelet counts, albumin, and metastatic site distribution (all *p* ≥ 0.203); a higher proportion of smokers was observed in the second-line group (63.8% vs. 45.2%, *p* = 0.041), the only baseline characteristic that differed significantly. Baseline characteristics are presented in Table 1. All patients had bladder primary tumors and de novo metastatic (stage IV) disease, so tumor location and clinical stage were uniform across the cohort and did not differ between groups. The duration of first-line therapy, defined as the interval from first-line initiation to documented progression, did not differ significantly between groups (median 6.9 [IQR 5.0–9.8] months in the second-line group versus 7.4 [IQR 5.5–9.0] months in the no-second-line group; *p* = 0.60). Among the 80 patients who received second-line chemotherapy, paclitaxel was the most commonly administered regimen (60 patients, 75.0%), followed by vinflunine (10, 12.5%), docetaxel (7, 8.8%), and MVAC (3, 3.8%) (Table 2).

### 3.2. Overall Survival

**Figure 1:**
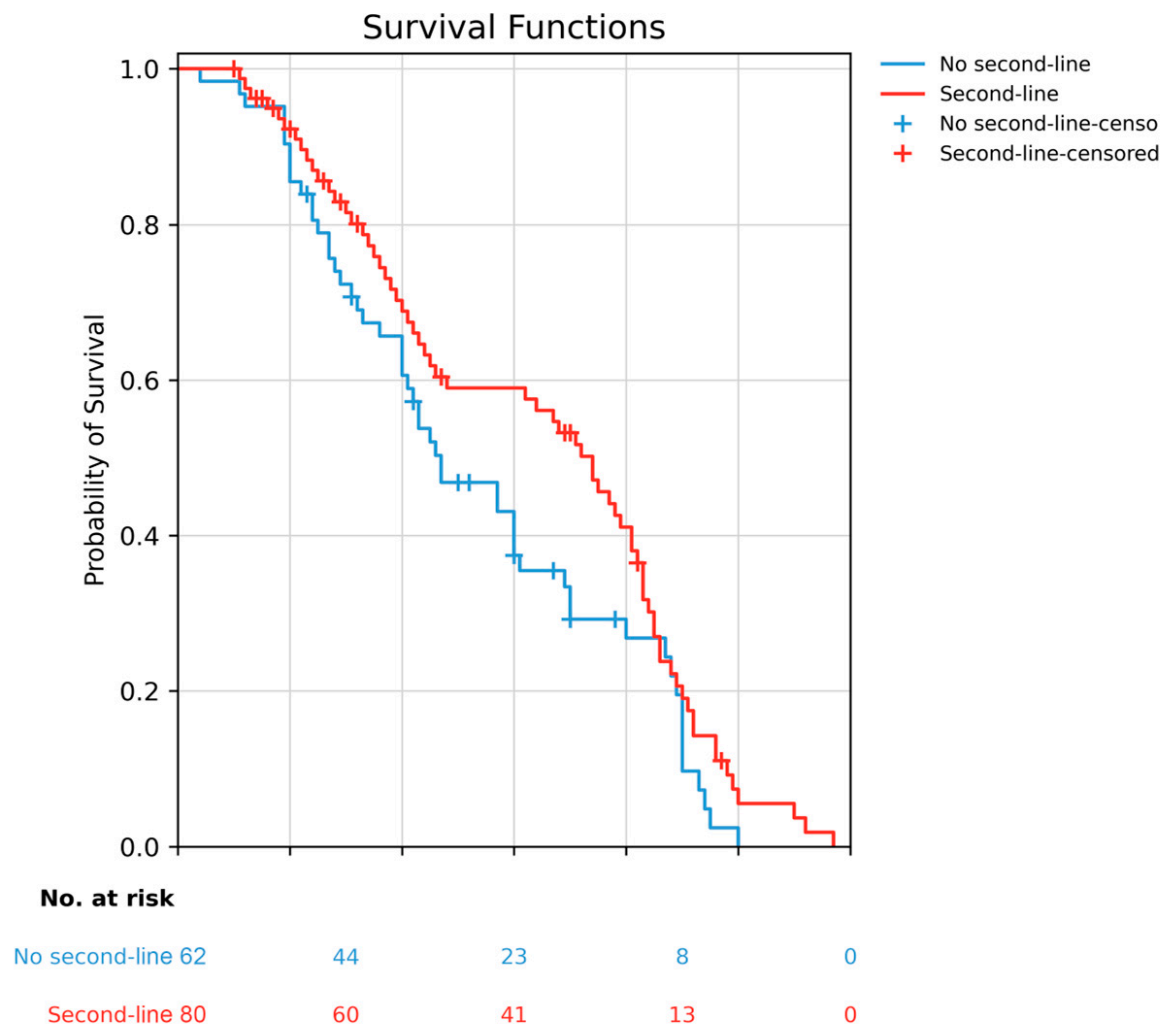
Overall survival by second-line treatment status, from progression on first-line therapy. Footnote: OS was estimated using the Kaplan–Meier method and compared using the log-rank test (*p* = 0.064). Shaded bands represent 95% confidence intervals.

At analysis, 120 of 142 patients (84.5%) had died; the censoring rate was low (15.5%), so the reverse Kaplan–Meier median follow-up could not be reliably estimated; median observed follow-up among the 22 censored patients was 4.5 months (IQR 2.4–6.9). Median OS was 7.4 months (95% CI 4.5–8.1) in the second-line group versus 4.7 months (95% CI 4.0–6.0) in the no-second-line group (log-rank *p* = 0.064), with 6-month survival rates of 59% and 37%, respectively (Table 3, Figure 1). In univariate Cox analysis, second-line treatment showed a non-significant association with reduced risk of death (HR 0.700; 95% CI 0.483–1.013; *p* = 0.059).

### 3.3. Multivariable Analysis and Robustness Across Bias-Adjustment Methods

In multivariable Cox regression, second-line chemotherapy was independently associated with improved OS (aHR 0.620; 95% CI 0.423–0.907; *p* = 0.014). ECOG ≥ 2 was the strongest independent predictor of death (aHR 3.881; 95% CI 1.773–8.496; *p* = 0.001), and lower albumin was independently associated with worse survival (aHR 0.671 per g/dL; 95% CI 0.482–0.934; *p* = 0.018); age, smoking, and GFR were not independently associated with OS (Table 4). Model discrimination was modest (C-index 0.639). The proportional-hazards assumption was satisfied for all covariates (Schoenfeld test, all *p* > 0.19; Table 4). In the sensitivity analysis restricted to patients with ECOG 0–1 (*n* = 129), the association between second-line treatment and OS remained significant and was essentially unchanged in magnitude (aHR 0.589; 95% CI 0.399–0.870; *p* = 0.008; Supplementary Table S2). Substituting the Bellmunt risk score for its components yielded a directionally and statistically consistent estimate for second-line treatment (aHR 0.656; 95% CI 0.449–0.957; *p* = 0.029), although the Bellmunt score itself was not independently significant (aHR 1.250 per point; 95% CI 0.968–1.614; *p* = 0.087; Supplementary Table S2).

**Figure 2:**
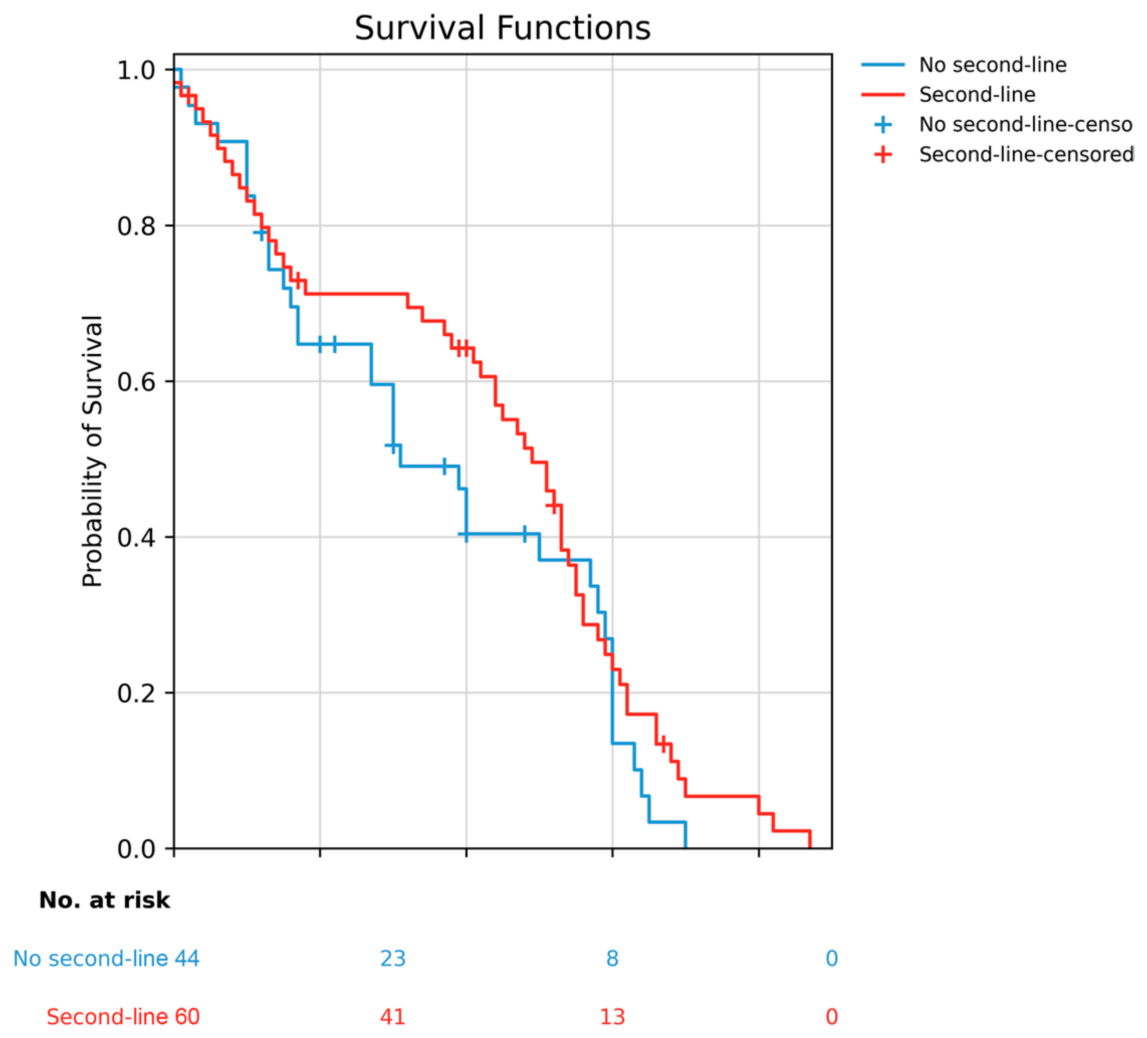
Landmark analysis restricted to patients alive at 3 months. Footnote: OS from the 3-month landmark was estimated using the Kaplan–Meier method and compared between patients who ever received second-line chemotherapy and those who did not, using the log-rank test (*p* = 0.197). Patients who died or were censored before the landmark (*n* = 38) were excluded from this analysis.

The association was then tested against four analyses designed specifically to address confounding and immortal-time bias (Table 5). After stabilized IPTW, covariate balance was excellent for all seven covariates (SMD < 0.1; Supplementary Table S1, Supplementary Figure S1); the association with OS remained significant (HR 0.648; 95% CI 0.444–0.947; *p* = 0.025). In 1:1 propensity-score matching (50 matched pairs, *n* = 100), the point estimate was materially unchanged (HR 0.645; 95% CI 0.416–1.001) but narrowly missed statistical significance (*p* = 0.051). Among the 80 patients who received second-line chemotherapy, the interval from progression to second-line initiation, calculated from recorded calendar dates, was short (median 0.4 months; IQR 0.2–0.7; range 0.03–1.7). A time-dependent Cox model, which classifies this interval as unexposed person-time and retains the full cohort without discarding early events, confirmed the association (HR 0.632; 95% CI 0.432–0.926; *p* = 0.019), closely consistent with the primary multivariable estimate. In the 3-month landmark analysis (104 patients alive at 3 months; 38 patients who died or were censored before 3 months were excluded), median OS from the landmark was 4.9 months (95% CI 4.1–5.4) with second-line treatment versus 3.1 months (95% CI 1.7–5.7) without (log-rank *p* = 0.197; HR 0.743; 95% CI 0.482–1.147; *p* = 0.180) (Figure 2); this was clearly non-significant. Sensitivity analyses using 2- and 4-month landmarks yielded materially unchanged estimates (HR 0.690, 95% CI 0.466–1.020, *p* = 0.063 at 2 months; HR 0.645, 95% CI 0.404–1.030, *p* = 0.066 at 4 months; Supplementary Table S3), indicating that the specific landmark time point did not drive this result. With 120 observed events, the study had approximately 49% power to detect the hazard ratio observed in the univariate analysis (0.70); approximately 251 events would have been required for 80% power.

## 4. Discussion

In this retrospective analysis of 142 patients with metastatic urothelial carcinoma, second-line chemotherapy was associated with a numerically longer median OS (7.4 vs. 4.7 months) that approached but did not reach conventional statistical significance by log-rank test (*p* = 0.064). In the primary multivariable model, this association was statistically significant (aHR 0.620; *p* = 0.014) and remained essentially unchanged in a sensitivity analysis restricted to patients without severe functional impairment (aHR 0.589; *p* = 0.008) and in a model substituting the composite Bellmunt risk score (aHR 0.656; *p* = 0.029). The association remained significant after IPTW (HR 0.648; *p* = 0.025) and, notably, after a time-dependent Cox model that treated second-line chemotherapy as a time-varying covariate (HR 0.632; *p* = 0.019)—a widely recommended approach to immortal-time bias, since it retains the full cohort and classifies pre-treatment person-time as unexposed without discarding early events or patients (other approaches, such as marginal structural models or the g-formula, exist but were not applied here). Two analyses did not reach statistical significance: propensity-score matching (HR 0.645; *p* = 0.051) and the 3-month landmark analysis (HR 0.743; *p* = 0.180). In both, the point estimate remained below 1.0 and in the same direction as the other analyses, and across the 2-, 3-, and 4-month landmarks the estimates were consistent (HR 0.690, 0.743, and 0.645, respectively), indicating a stable effect direction even where statistical significance was not confirmed.

The time-dependent Cox model, generally regarded as the most rigorous approach to immortal-time bias, confirmed the association and agreed closely with the primary multivariable estimate; the conventional Cox, IPTW, and propensity-score-matched analyses share the same baseline-exposure definition and therefore the same potential time-related bias, and are not independent confirmations. Against this, the borderline propensity-score matching result and the non-significant landmark analysis are informative. Unlike the landmark approach, the time-dependent Cox model discards no patients or person-time, and its close agreement with the primary multivariable estimate argues against immortal-time bias as the principal explanation for the primary finding. This convergence is consistent with the short interval from progression to second-line initiation observed in this cohort (median 0.4 months), which limits the amount of guaranteed survival time available to bias a naive comparison. The landmark analysis’s divergence is therefore more plausibly explained by its substantial loss of patients and events (38 of 142 patients, 26.8% of the cohort, were excluded because they died or were censored before 3 months) than by a bias uniquely corrected by that method; the borderline propensity-score matching result is consistent with the same reduction in sample size. The stability of the landmark estimate across 2-, 3-, and 4-month cut-points (Supplementary Table S3) suggests this pattern is not an artifact of the specific landmark time chosen. We present all seven estimates transparently (Table 5) rather than selecting the analysis most favorable to a positive conclusion, and acknowledge that residual immortal-time bias, though now less likely given the time-dependent Cox result, cannot be entirely excluded.

These results should be interpreted within the rapidly evolving landscape of metastatic urothelial carcinoma. The EV-302/KEYNOTE-A39 trial established first-line enfortumab vedotin plus pembrolizumab as superior to platinum-based chemotherapy, nearly doubling median OS (31.5 vs. 16.1 months; HR 0.47), and this combination is now the standard of care [16]. Antibody–drug conjugates and targeted therapy in biomarker-selected patients have further expanded subsequent-line options. Against this backdrop, our findings on conventional second-line chemotherapy are best understood as a benchmark for its efficacy in settings where these newer agents are not yet accessible, rather than as evidence against pursuing novel agents where they are available. Whereas the pivotal vinflunine and taxane studies established the activity of second-line chemotherapy in selected clinical-trial populations, the present analysis quantifies its survival contribution in an unselected real-world cohort while formally separating treatment effect from selection, using multiple parallel bias-adjustment methods—evidence that complements, rather than duplicates, the existing trial literature.

The median overall survival observed in our cohort—7.4 months in treated and 4.7 months in untreated patients—is broadly concordant with the modest outcomes historically reported for second-line cytotoxic therapy, such as the 6.9 months seen with vinflunine in its pivotal phase III trial [7]. An important consideration specific to our setting concerns switch-maintenance immunotherapy: in the JAVELIN Bladder 100 trial, avelumab maintenance significantly prolonged OS compared with best supportive care (median 23.8 vs. 15.0 months; HR 0.76), and it is a reimbursed option in our country [17,18]. Patients receiving avelumab maintenance were deliberately excluded to preserve cohort homogeneity; our cohort therefore represents patients managed with a chemotherapy-based sequence without maintenance immunotherapy, and our findings should not be extrapolated to the broader population. Recent real-world analyses of avelumab maintenance have similarly cautioned that favorable survival estimates in maintenance-treated cohorts are strongly influenced by selection effects and immortal-time bias, underscoring the general importance of the multi-method analytical approach taken here [24,25,26].

The consistently strong and stable effect of ECOG performance status across every model—its magnitude and significance did not vary meaningfully across the primary, sensitivity, weighted, matched, or landmark analyses—is in keeping with established prognostic models in this disease. Performance status is a core component of both the Bajorin score, developed in the first-line cisplatin setting, and the Bellmunt score, developed for the post-platinum second-line setting [19,20]. Notably, ECOG performance status was well balanced between treatment groups at baseline (ECOG ≥ 2 in 11.3% vs. 6.5%; *p* = 0.490) and achieved excellent balance after IPTW (Supplementary Table S1), suggesting that its strong prognostic effect reflects a genuine, independent association with survival rather than confounding by treatment selection. This reinforces the importance of thorough functional assessment when considering second-line chemotherapy.

This study has several limitations. Its retrospective, non-randomized design renders it susceptible to residual confounding despite multivariable adjustment and propensity-score methods; unmeasured or incompletely captured factors—including comorbidity burden beyond ECOG, molecular tumor characteristics, treating physician discretion, institutional variation in supportive care across the seven participating centers, and socioeconomic factors affecting treatment access—could not be adjusted for and may have influenced both the decision to offer second-line chemotherapy and survival independently of that decision. The landmark analysis and propensity-score matching both address specific biases (immortal-time bias and confounding, respectively) at a considerable cost in sample size and power, and their non-significant or borderline results should be weighed against this trade-off; the stability of the landmark point estimate across 2-, 3-, and 4-month landmark points (Supplementary Table S3) provides some reassurance that the specific landmark choice did not drive its divergence from the other analyses. Across the analyses, the point estimates for second-line chemotherapy were consistently directionally concordant (hazard ratios below 1.0); where individual analyses did not reach statistical significance, this occurred in the analyses most affected by reduced sample size, and these results are therefore best interpreted as non-confirmatory rather than as evidence against a treatment effect. Second-line regimens were heterogeneous (Table 2), and the cohort was not large enough to compare individual agents reliably. The single-country setting and the exclusion of patients receiving maintenance immunotherapy limit generalizability. During the study period, antibody–drug conjugates and targeted therapies were not reimbursed and therefore not accessible, so the second-line options captured were confined to conventional cytotoxic chemotherapy; findings cannot be extrapolated to contemporary practice using these agents. Finally, molecular biomarkers were not systematically available. Prospective studies incorporating novel agents and maintenance strategies, with complete treatment-timing and regimen data, are warranted.

In conclusion, in this real-world cohort managed without maintenance immunotherapy, second-line chemotherapy was independently associated with improved overall survival in the primary multivariable model and, most importantly, in a time-dependent Cox model specifically designed to address immortal-time bias without discarding patients or person-time; the conventional Cox, IPTW, and propensity-score-matched analyses were directionally concordant but share a common time-related exposure definition and are not independent confirmations. The landmark analysis and, narrowly, propensity-score matching did not reach significance, plausibly reflecting reduced statistical power after exclusion of patients rather than a materially different underlying effect. ECOG performance status was, in parallel, a robust and consistent independent predictor of outcome across every analytical approach. In many middle-income countries, including Türkiye, access to enfortumab vedotin and other novel agents remains limited, and conventional chemotherapy continues to be widely used in routine practice; these findings support its continued use while highlighting the need to integrate antibody–drug conjugates and immunotherapy-based approaches where access permits. Larger, prospective studies with complete treatment-timing data are needed to confirm the magnitude of benefit and resolve the discordance with the landmark estimate.

## Figures and Tables

**Table 1:** Baseline characteristics according to second-line treatment status.

Characteristic	Second-Line (*n* = 80)	No Second-Line (*n* = 62)	*p*
Age, years, median (IQR)	64.0 (57.5–72.5)	66.0 (60.0–71.0)	0.557
Male sex, n (%)	65 (81.3)	56 (90.3)	0.203
Current/former smoker, n (%)	51 (63.8)	28 (45.2)	0.041
ECOG ≥ 2, n (%)	9 (11.3)	4 (6.5)	0.490
Albumin, g/dL, median (IQR)	3.7 (3.3–4.0)	3.8 (3.4–4.1)	0.210
eGFR, mL/min/1.73 m^2^, median (IQR)	73.5 (53.5–90.0)	65.0 (46.2–87.8)	0.443
Hemoglobin, g/dL, median (IQR)	11.0 (9.3–12.9)	11.4 (10.0–13.0)	0.304
Lymphocyte, ×10^9^/L, median (IQR)	1.5 (1.1–2.0)	1.6 (1.2–2.2)	0.358
Platelet, ×10^9^/L, median (IQR)	252.5 (210.0–356.5)	287.0 (224.5–372.5)	0.317
Liver metastasis, n (%)	19 (23.8)	14 (22.6)	1.000
Lung metastasis, n (%)	43 (53.8)	40 (64.5)	0.263
Bone metastasis, n (%)	35 (43.8)	32 (51.6)	0.446
Brain metastasis, n (%)	6 (7.5)	2 (3.2)	0.466
First-line duration, months, median (IQR)	6.9 (5.0–9.8)	7.4 (5.5–9.0)	0.60

Footnote: Values are n (%) unless otherwise indicated. Continuous variables are presented as median (IQR) and compared using the Mann–Whitney U test; categorical variables were compared using the chi-square or Fisher’s exact test. Abbreviations: ECOG, Eastern Cooperative Oncology Group; eGFR, estimated glomerular filtration rate.

**Table 2:** Second-line chemotherapy regimens administered (*n* = 80).

Regimen	*n*	%
Paclitaxel	60	75.0
Vinflunine	10	12.5
Docetaxel	7	8.8
MVAC	3	3.8

Footnote: Regimen was recorded for all 80 patients who received second-line chemotherapy; no patient received more than one regimen concurrently. Abbreviation: MVAC, methotrexate, vinblastine, doxorubicin, and cisplatin.

**Table 3:** Overall survival outcomes according to second-line treatment status.

	Second-Line (*n* = 80)	No Second-Line (*n* = 62)	*p*
Deaths, n (%)	67 (83.8)	53 (85.5)	—
Median OS, months (95% CI)	7.4 (4.5–8.1)	4.7 (4.0–6.0)	0.064 *
6-month survival, %	59	37	—
Univariate HR (95% CI)	0.700 (0.483–1.013)	reference	0.059

Footnote: * Log-rank *p*-value; the univariate HR *p*-value is from Cox regression. Abbreviations: CI, confidence interval; HR, hazard ratio; OS, overall survival.

**Table 4:** Multivariable Cox regression for overall survival (ECOG modelled as 0–1 vs. ≥2; *n* = 142, 120 events, C-index 0.639).

Covariate	aHR	95% CI	*p*	PH Test *p* ^a^
Second-line chemotherapy	0.620	0.423–0.907	0.014	0.888
ECOG ≥ 2 (vs. 0–1)	3.881	1.773–8.496	0.001	0.866
Albumin (per g/dL)	0.671	0.482–0.934	0.018	0.194
Age (per year)	0.988	0.968–1.007	0.219	0.447
Smoking (yes vs. no)	1.059	0.731–1.534	0.762	0.906
eGFR (per unit)	1.004	0.997–1.011	0.215	0.361

Footnote: ^a^ Schoenfeld residual test *p*-value for the proportional-hazards assumption; *p* > 0.05 indicates the assumption was satisfied for that covariate. Abbreviations: aHR, adjusted hazard ratio; CI, confidence interval; ECOG, Eastern Cooperative Oncology Group; eGFR, estimated glomerular filtration rate; PH, proportional hazards.

**Table 5:** Comparison of hazard ratio estimates for second-line chemotherapy across analytical approaches.

Analysis	*n*	HR (95% CI)	*p*	Purpose
Univariate Cox	142	0.700 (0.483–1.013)	0.059	Unadjusted
Multivariable Cox	142	0.620 (0.423–0.907)	0.014	Primary model
Time-dependent Cox	142	0.632 (0.432–0.926)	0.019	Immortal-time bias, no data loss
IPTW (stabilized)	142	0.648 (0.444–0.947)	0.025	Confounding, all measured covariates
Bellmunt-score model	142	0.656 (0.449–0.957)	0.029	Composite risk adj.
Sensitivity (ECOG 0–1 only)	129	0.589 (0.399–0.870)	0.008	Excludes ECOG ≥ 2
Propensity-matched (50 pairs)	100	0.645 (0.416–1.001)	0.051	Confounding, supportive
3-month landmark	104	0.743 (0.482–1.147)	0.180	Immortal-time bias

Footnote: All models estimate the hazard ratio for second-line chemotherapy versus no second-line chemotherapy on overall survival. IPTW used stabilized weights with robust standard errors; propensity-score matching used 1:1 nearest-neighbor matching with a caliper of 0.2 SD of the logit propensity score. Abbreviations: CI, confidence interval; HR, hazard ratio; IPTW, inverse probability of treatment weighting.

## Data Availability

The datasets generated and analyzed during the current study are available from the corresponding author on reasonable request. The data are not publicly available due to ethical restrictions and patient confidentiality.

## Supplementary Materials

The following supporting information can be downloaded at: https://www.mdpi.com/article/10.3390/curroncol33090529/s1; Figure S1: Love plot of covariate balance before and after inverse probability of treatment weighting; Table S1: Standardized mean differences before and after inverse probability of treatment weighting; Table S2: Secondary multivariable Cox models (Bellmunt risk score model and ECOG 0–1 sensitivity analysis); Table S3: Landmark sensitivity analysis at 2, 3, and 4 months.
