# Supplementary material for: Real-World Outcomes of Second-Line Chemotherapy in Metastatic Urothelial Carcinoma
Source: Curr Oncol. 2026 Sep 2;33(9):529. doi: 10.3390/curroncol33090529 (PMC13605251; doi:10.3390/curroncol33090529)
Supplement: Supplementary file 1 [file curroncol-33-00529-s001.zip › curroncol-4489289-SI.pdf]

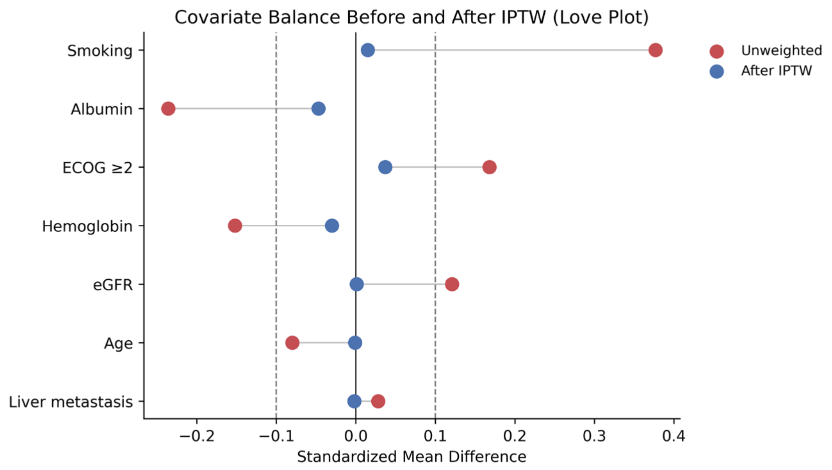

Supplementary Figure S1. Love plot of covariate balance before and after inverse probability of treatment weighting.

Footnote: Points show the standardized mean difference (SMD) for each covariate between the second-line and no-second-line groups before (red) and after (blue) IPTW; dashed lines mark the conventional |SMD| = 0.1 threshold for good balance. All seven covariates achieved good balance after weighting. Numeric values are provided in Supplementary Table S1.

Supplementary Table S1. Standardized mean differences before and after inverse probability of treatment weighting.

**Covariate**	**SMD, unweighted**	**SMD, after IPTW**
ECOG ≥2	0.168	0.037
Age	−0.080	−0.001
Smoking	0.377	0.015
Albumin	−0.236	−0.047
eGFR	0.121	0.001
Hemoglobin	−0.152	−0.030
Liver metastasis	0.028	−0.002

Footnote: SMD <0.1 indicates good covariate balance between second-line and no-second-line groups. Abbreviation: IPTW, inverse probability of treatment weighting.

Supplementary Table S2. Secondary multivariable Cox models (Bellmunt risk score model and ECOG 0–1 sensitivity analysis).

**Covariate**	**Bellmunt-score model[b] aHR (95% CI)**	**p**	**ECOG 0–1 sensitivity[c] aHR (95% CI)**	**p**
Second-line chemotherapy	0.656 (0.449–0.957)	0.029	0.589 (0.399–0.870)	0.008
Bellmunt score (per point)	1.250 (0.968–1.614)	0.087	—	—
Age (per year)	0.988 (0.968–1.008)	0.239	0.986 (0.966–1.007)	0.182
Smoking (yes vs no)	1.164 (0.808–1.676)	0.415	1.072 (0.737–1.560)	0.717
Albumin (per g/dL)	0.756 (0.528–1.082)	0.126	0.680 (0.483–0.958)	0.028
eGFR (per unit)	1.006 (0.999–1.013)	0.099	1.004 (0.997–1.011)	0.286

Footnote: [b]Model substituting the Bellmunt risk score (ECOG >0, hemoglobin <10 g/dL, liver metastasis; range 0–3) for its individual components (n=142, 120 events, C-index 0.623). [c]Model restricted to patients with ECOG 0–1 (n=129, 112 events, C-index 0.619).

Supplementary Table S3. Landmark sensitivity analysis at 2, 3, and 4 months.

Landmark	n	HR (95% CI)	p	Log-rank p
2 months	126	0.690 (0.466–1.020)	0.063	0.068
3 months (primary)	104	0.743 (0.482–1.147)	0.180	0.197
4 months	89	0.645 (0.404–1.030)	0.066	0.072

Footnote: Patients who died or were censored before the respective landmark time were excluded from that row. HR estimated by Cox regression from the landmark time; log-rank p from the corresponding Kaplan–Meier comparison. The 3-month landmark was used as the primary analysis (Table 5, Figure 2).
